# Satisfaction with life and opioid dependence

**DOI:** 10.1186/1747-597X-3-2

**Published:** 2008-01-28

**Authors:** Jason Luty, Sujaa Mary Rajagopal Arokiadass

**Affiliations:** 1 Department of Psychiatry, South Essex Partnership NHS Trust, Southend on Sea, Essex, UK; 2 Department of Forensic Psychiatry, South Essex Partnership NHS Trust, Southend on Sea, Essex, UK

## Abstract

**Background:**

Serious substance misuse and dependence is widely seen as damaging to an individual and to society in general. Whereas the medical and society effects of substance misuse are widely described, some commentators suggest substance misuse may be an "alternative lifestyle".

**Aim:**

To assess general life satisfaction amongst treatment-seeking people with substance dependence.

**Methods:**

The Satisfaction With Life Scale (SWLS) was administered to a sample of opioid-dependent people receiving substitute medication.

**Results:**

105 subjects and 105 age-sex matched subjects in a comparison group completed the questionnaire. The mean SWLS score was 7.12 (SD = 10.6; median = 6) for patients compared to 22.6 (SD = 6.8) in the comparison group. (Two sided p < 0.0001; Median difference = -13.5; Wilcoxon signed rank test.)

**Conclusion:**

The study used a validated instrument and objective reports to confirm significantly higher rates of dissatisfaction with life among opioid dependent people in treatment when compared to members of the general population.

## Introduction

The British Government commissioned several reports in the 1950s in regard to what are often termed "victimless crimes". Recommendations from these reports into prostitution, abortion and homosexuality were gradually introduced (with repeal of the relevant legislation). However the recommendations regarding more liberal legislation on illicit drug use (The Wootton Report, 1969) was never fully introduced [1,2]. Consideration was given at both committees that illicit drug use could be regarded as an alternative lifestyle rather than a criminal deviance or disease. If this were the case, it would be possible that illicit drug users might have the same overall satisfaction with their life as other members of the public. (Clearly, however, social disapproval and legal sanctions might cause lifestyle problems that were unrelated to the acquisition and use of addictive drugs.) One question facing many authorities remains, can illicit drug use be regarded as a valid, although reckless, lifestyle choice (much like rock climbing or motor sport)? To better inform policy decisions on substance misuse a survey was conducted of satisfaction with life of people in treatment for illicit drug problems and members of the public.

Serious substance misuse and dependence is widely seen as damaging to an individual and to society in general [3-8]. Whereas the medical and societal effects of substance misuse are widely described, some commentators suggest substance misuse may be an "alternative lifestyle" [9,10]. Emotional and behavioural problems, including delinquency, truancy and hyperactivity, have repeatedly been found to be associated with and predict substance misuse [11-13].

The aim of the current research was to use a validated instrument to assess general life satisfaction amongst treatment-seeking people with substance (opioid) dependence.

## Methods

Participants were approached by researchers at three drug and alcohol services in South East England. Participants were included who were dependent on illicit opioids drugs in receipt of substitute medication. All patients subject to review by medical staff were approached and information sheets were also distributed at the reception of the clinic. The services currently provide substitute prescriptions to approximately 600 patients. The inclusion criteria were: currently receiving treatment for opioid dependence and ability to give written informed consent. There were no specific exclusion criteria. Illicit drug dependence was confirmed using the Minnesota Student Survey Screening [14]. Comparison group subjects were age and sex matched to within 5 years. Comparison group subjects were recruited from the general UK population from a database created as part of another study [15]. They were recruited by direct mail shots sent to addresses at random and newspaper advertisements throughout the UK. The project was approved by the local research ethics committee. All participants completed the Satisfaction With Life Scale [16]. However only patients had urine drug screens performed. Patients also completed some questions on their involvement with the police, school engagement and clinical information such as the duration of illicit drug use. This information was not obtained for the non-drug using comparison group.

The Satisfaction With Life Scale (SWLS; [16,17]) is an extensively validated 6-item self-completion instrument (score 1 to 7). Responses are scored on a 6-point Likert-type scale yielding a maximum overall score range from 5–35.

## Results

One hundred and five subjects completed the questionnaire. These were paired with 105 age and sex-matched comparison subjects drawn from the general UK population. Comparison subjects were matched by age to within 5 -years. The 105 patients included in the study had a mean age of 33.3 (SD = 12.8) years; 80% were male; 10% were in paid employment; 96% described themselves as white British. Comparison subjects had a mean age of 34.8 (SD = 18.6) years; 76% were in paid employment. In practice, the majority of the patients were receiving long-term prescriptions (in excess of 2 years) although many were attempting to gradually detoxify. 45% were in receipt of buprenorphine prescriptions, the remainder received methadone. The mean age at first heroin use was 21.3 (SD = 7) years and they reported using opiates regularly for 14.2 (SD = 9) years.

The mean SWLS score was 7.12 (SD = 10.6; median = 6) for patients compared to 22.6 (SD = 6.8) in comparison subjects. (Two sided p < 0.0001; Median difference = -13.5; Wilcoxon signed rank test.)

The SWLS scores for the comparison subjects were not significantly different from those from the original validation study. (The mean score was 23.5 (SD = 6.43) in 176 US undergraduates in the original validation report; [16]).

Forty per cent of patients were expelled from school while 32 (64%) received no formal qualifications – this is loosely comparable to not completing US high school. Patients obtained a mean of 1.88 (SD = 2.62; median = 0; n = 18) GCSEs or O-levels (qualification obtained at the age of 16 in the UK). The mean age of first contact with the police in patients was 13.3 (SD = 3.0 n = 48) years. The mean age of first use any illicit drug use in patients was 16.1 (SD = 4.5) years. The mean age of first use of heroin or cocaine in patients was 19.5 (SD = 5.3) years. Ninety six per cent of patients reported problems with the police in adolescence.

## Discussion

The report clearly shows that opioid dependent people who are in treatment have much lower levels of satisfaction than members of the comparison group. Happiness and "satisfaction" with life are global concepts with philosophical and psychological components [16-18]. The results refer to a treatment seeking population – although these are likely to be representative of those people dependent on illicit opiates in the UK (the majority of who are in contact with treatment services [19]), this would not represent those who tend not to access treatment including people who use infrequently or those who use other illicit drugs such as cannabis or stimulants.

There are many potential determinants of satisfaction with life. These include personality, social expectations, socio-economic factors especially relative deprivation, relationships with significant others (neighbours, parents and children), physical and psychological health, accommodation, employment and problem with authority [20,21]). Moreover there is overwhelming evidence of the damaging effects of illicit drug dependence on both the physical and mental health of users and also on their relationships and social functioning [3,9]. Three potential explanations can be cited. Firstly, opiate dependence leads to chronic mental health problems and physical illness that directly cause dis-satisfaction ("dis-ease"). Secondly, opiate dependence causes secondary social and relationship problems that prevent people achieving their desired goals (e.g. criminality restricts employment; substance misuse damages relationships with family and significant others). Thirdly, it remains possible opiate dependent people have behavioural and psychological traits that prevent them achieving "happiness" – that is these people would remain dissatisfied with life regardless of whether they became substance abusers or not. Whereas it is extremely difficult to disentangle these competing theories, the results presented here clearly show that people with substance misuse problems, even those in treatment, are generally dissatisfied with life and "unhappy".

In relation to the second hypothesis (that dissatisfaction results from secondary social problems resulting indirectly from substance use and acquisition), there are very many possible causes including predisposing factors (history of depression, conduct disorder, poverty in the home) and current concomitant factors (lack of employment, poverty, health, legal sanctions). Some of these may be amenable to intervention and many drug treatment services aim to provide these, including the services from which the patients were recruited. These include advice on benefits and employment and assistance with housing as well as treatment for depression. A comprehensive model for holistic assessment and treatment of substance using people is described by UK government guidelines and are enacted, at least in theory, in all NHS facilities [3,19]. Despite these attempts at resolving the many social difficulties, the patients in treatment remained dissatisfied with life. There has been a longstanding debate regarding the potentially damaging effects of rendering addictive drugs illegal and requests for decriminalizing drugs [1,2]. However any actions in this respect are based primarily on political rather than scientific grounds and these are probably unlikely in the current political climate [22].

## Strengths and limitations

The Satisfaction with Life Scale is a well-validated questionnaire that has been compared to several other instruments and has good psychometric properties [16-18].

Opioid-dependent people may be motivated to seek treatment as a result of dissatisfaction with life. It remains possible that a proportion of opiate users are satisfied with life and do not seek treatment. However other research suggests that at around 80% of illicit opioid users have been in contact with treatment services and at least half are in contact at any one time [23,24].

## Conclusion

The study used a validated instrument and objective reports to confirm significantly higher rates of dissatisfaction with life among opioid dependent people in treatment when compared to members of the general population.

## Competing interests

The author(s) declare that they have no competing interests.

## Authors' contributions

Both authors were fully and actively involved in all parts of the project including design, data collection, analysis and manuscript preparation. All authors read and approved the final manuscript.
